# Congenital hypopituitarism: how to select the patients for genetic analyses

**DOI:** 10.1186/s13052-018-0484-y

**Published:** 2018-04-06

**Authors:** Giuseppe Crisafulli, Tommaso Aversa, Giuseppina Zirilli, Filippo De Luca, Romina Gallizzi, Malgorzata Wasniewska

**Affiliations:** Department of Human Pathology in Adulthood and Childhood, University of Messina, Via Consolare Valeria, 98124 Messina, Italy

**Keywords:** Causative gene mutations, Combined pituitary hormone deficiency, HESX1 gene, LHX3 and LHX4 genes, Multiple pituitary hormone deficiency, POU1F1 gene, PROP1 gene

## Abstract

Aim of this commentary is to analyze the relationships between genotypic bases and phenotypic expression of congenital “multiple pituitary hormone deficiency” (MPHD) syndrome and to indicate some reliable criteria for selecting the patients who should undergo genetic analyses in order to clarify the etiology of their disorder.

On the basis of the most recent reports on this topic, it is possible to infer that: 1) in only few patients with congenital MPHD it is possible to detect a causative gene mutation; 2) therefore, it is fundamental to define some criteria for selecting the patients who should undergo genetic analyses; 3) such inclusion criteria should be based on the overall evaluation of hormonal clinical and neuroradiological phenotype; 4) it is crucial to consider whether the cases are sporadic or familial, since the probability of finding a causative gene mutation is distinctly higher in familial cases; 5) for PROP1 gene it is also important to consider the geographical origin of the patients, because this mutation is much more frequent in some ethnic groups.

## Background

GH deficiency (GHD) is a relatively uncommon pathological condition, which should always be considered in the diagnostic work-up of a child with growth retardation and no other identified causes. Classic GHD has been reported as frequently as 1 in 4000 children [1], but such prevalence rate may be even higher when acquired GHD, due to inflammatory or neoplastic endocranial diseases or to head and neck irradiation, is also taken into consideration.

Classic GHD has been considered for a long time as an idiopathic condition, secondary to decreased hypothalamic GHRH stimulation. In the last years, nevertheless, the exponentially increasing advances in genetic studies have allowed to clarify that some patients, who were previously classified as having an idiopathic GHD, may show a specific molecular defect in the genes that are involved in the regulation of pituitary organogenesis or function [2]. Such a novel diagnostic approach, based on genetic analyses, might involve both the patients with familial or sporadic hypopituitarism and those with isolated GHD or multiple pituitary hormone deficiency (MPHD) [2]. However, in a current environment of limited economic resources, it is essential to establish inclusion criteria for specific genetic analyses [3].

The main genetic causes of some forms of congenital hypopituitarism have been recently systematically reviewed and it was concluded that fully penetrant mutations within known genes may be detected in only 20% of familial cases, whilst the frequency of mutations in sporadic cases is even much lower [3]. This might explain why, in most cases, the etiology of congenital hypopituitarism remains uncertain. However, novel genetic determinants of pituitary disorders will be probably identified in the subsequent years, with the availability of next generation sequencing technology in the diagnostic work-up of children with documented GHD [2].

Aim of the present commentary is to analyze the relationships between genotypic bases and phenotypic expression of congenital MPHD syndrome and to identify some reliable criteria for selecting the MPHD patients who should undergo genetic analyses in order to clarify the etiology of their disorder.

### Clinical and neuroradiological features of congenital MPHD syndrome

This condition is very often sporadic and only rarely familial and is preponderant in male sex [3]. It is characterized, by definition, by the impaired secretion of GH and one or more other pituitary hormones, as a consequence of either hypothalamic or pituitary disease.

According to the results of the recent study by De Rienzo et al. [3], covering 144 Italian patients with congenital MPHD, GHD is always present, whilst gonadotropin and TSH deficiencies are not constant and are encountered, respectively, in 82 and 81.3% of cases. ACTH deficiency (57.6%) and diabetes insipidus (3.5%) are detectable in more limited percentages of patients [3].

Clinical picture may present very early, at birth or shortly afterwards, with varying combinations of hypoglycemia and prolonged cholestatic neonatal jaundice in both sexes and microphallus and/or bilateral cryptorchidism in boys. In most cases, however, diagnosis is suspected during the first years of life, owing to the finding of a severe and progressive growth and bone age retardation [3].

Extra-endocrine clinical manifestations include craniofacial defects, such as septo-optic dysplasia, holoprosencephaly, corpus callosum aplasia, ocular abnormalities, limited neck rotation and short cervical spine [2, 3].

At intracranial magnetic resonance imaging (MRI), abnormalities of hypothalamus-pituitary region have been documented in an elevated percentage of the Italian patients with MPHD (82.4%). Pituitary hypoplasia is the neuroradiological alteration that is most frequently detected (61.4%), followed by either pituitary stalk abnormalities, or neuropituitary ectopia (20.2%), or both (14.0%). In 10.5% of the Italian patients with congenital MPHD, it is also possible to detect extra-pituitary neuroradiological abnormalities, such as septo-optic dysplasia or other midline defects [3].

A possible cause of early onset MPHD syndrome is anterior pituitary agenesis (APA), a condition that has been reported very rarely in literature [4, 5]. Compared to the MPHD children with visible adenohypophysis, those with APA seem to be at higher risk of a very early onset of clinical endocrine manifestations [5]. In fact, the prevalence of microphallus, neonatal hypoglycemia and neonatal cholestasis was found to be significantly higher in APA children than in the ones with other neuroradiological abnormalities [5]. Therefore, it was inferred that the finding of a very early and severe clinical picture of MPHD, in a term newborn, should arouse the clinical suspicion of APA [5], although it has to be underlined that an early onset hypopituitarism may also be associated with other neuroradiological abnormalities.

### Etiological role of the main gene mutations in MPHD syndrome

The study by De Rienzo et al. [3] analyzed the prevalence of mutations in the most important genes that are involved in the regulation of pituitary ontogenesis and function (PROP1, POU1F1, LHX3, LHX4 and HESX1) in Italian patients with MPHD. The results were also compared with those reported in patients from other countries.

Among the 5 genes which were specifically analyzed in the study population by De Rienzo et al. [3], the one that resulted to be most often mutated was PROP1: in 4/126 index cases analyzed (3.2%). These 4 patients did not exhibit a homogeneous clinical, endocrine and neuroradiological phenotype and 2/4 were sporadic, whilst the remaining 2 cases were familial. The overall PROP1 mutation rate was low in both sporadic and familial cases and similar to that recorded in other western European series [3]. PROP1 mutation rates were reported to be abnormally elevated in only few countries, such as Lithuania [6], Russia [7], Hungary [8], Portugal [9], Czech Republic [10] and Brazil [11], where total mutation rate for both sporadic and familial cases of MPHD ranges from 64.8% in Lithuania to 17.2% in Brazil. In the remaining countries, total mutation rate for PROP1 in cohorts with MPHD ranges from 0.8 to 3.7% [3]. In all geographical areas and ethnic groups, the relative frequency of PROP1 mutations is distinctly higher in familial cases than in sporadic patients: 48.5 vs 6.7% [3]. Another peculiarity of patients with PROP1 mutations is that they never exhibit alterations in the pituitary stalk or posterior hypophysis [12].

In the Italian cohort of De Rienzo et al. [3], another mutated gene was POU1F1: in 1/24 sporadic patients selected for this analysis, with an overall mutation rate of 4.2% for this gene. This patient exhibited, as expected, a deficiency of GH, TSH and prolactin, whereas ACTH production was normal and gonadotropin secretion could not be evaluated since, at the time of diagnosis, this girl was still pre-pubertal. At MRI this girl showed an anterior pituitary hypoplasia [3].

From the review of the literature, it is confirmed that mutation frequency of POU1F1 gene in sporadic cases with CPHD is very low also in other ethnic groups: 1.6% [3]. Its mutation frequency seems to be distinctly higher in individuals with at least one affected relative: 21.6% [3].

Finally, in the Italian study population of De Rienzo et al. [3], 1/133 patients with MPHD was found to bear a HESX1 mutation (0.8%), whereas none of the 101 analyzed patients was detected to bear a mutation of LHX3 and LHX4 genes. The only one patient with HESX1 mutation was a sporadic case with both MPHD (GH, TSH and ACTH) and pituitary stalk abnormality and ectopic posterior pituitary, but no septo-optic dysplasia at MRI [3].

In other MPHD cohorts, the only 3 mutations of HESX1 gene were found in sporadic patients, with an estimated frequency of 0.45%. Similarly, LHX3 mutations exhibited a global frequency of 0.5% (0.3% in sporadic cases and 11.1% in familial cases), whilst LHX4 mutations exhibited a global frequency of 0.9% (0.5% in sporadic cases and 18.8% in familial patients) [3].

Finally, in a very limited cohort of 4 MPHD children with neuroradiologically documented APA, we were not able to identify any mutations of PROP1, POU1F1, LHX3, LHX4, HESX1 and ISL1 genes [5].

### How to select the patients with congenital MPHD for genetic analyses

On the basis of the available evidence, it is possible to infer that genetic analyses should be preceded, in every case, by a pituitary MRI. In fact, although an abnormal neuroradiological picture is not necessarily associated with a specific gene mutation, it has to be considered that pituitary imaging can represent a further element to guide genetic screening of MPHD, together with hormonal pattern and extra-pituitary phenotype.

The hypothesis of a PROP1 mutation has to be carefully considered in a patient with GH, TSH, prolactin and gonadotropin deficiency, especially when no alterations in the pituitary stalk or posterior hypophysis are detected at MRI. A further indication for the PROP1 gene study is the finding of an intracranial pseudo-tumor, which may be detected in more than 40% of the patients with this mutation during the first decade of life [13, 14].

The hypothesis of a POU1F1 mutation is suggested by the finding of GH and prolactin deficiency, with severe growth retardation and variable degrees of TSH deficit.

A HESX1 mutation has to be taken into consideration in a patient with septo-optic dysplasia and variable degrees of pituitary hormone deficiency, ranging from isolated GHD to panhypopituitarism.

Mutations of LHX3 gene have to be suspected in individuals with variable degrees of anterior pituitary hormone deficiencies and extra-pituitary clinical manifestations, such as limited neck rotation and perceptive deafness.

The neuroradiological finding of a poorly developed sella turcica, in a child with congenital MPHD, should arouse the suspicion of a LHX4 mutation [3].

However, the endocrine phenotypes of all these mutations may be characterized by variable degrees of anterior pituitary hormone deficiency, ranging from isolated GHD to the complete failure of all anterior pituitary cell lineages and this complex hormonal picture may be furtherly complicated by a possible evolution of the endocrinological status over the life-span of the same patient [15].

Finally, another essential aspect which needs to be evaluated is whether MPHD syndrome is either sporadic or familial. In fact, all familial cases should undergo genetic analyses, since the probability of finding a causative mutation within any of the transcriptional factor genes is 63%, whilst such probability is very low in sporadic cases [15–17]. Therefore, in sporadic patients with MPHD, genetic analyses should be performed only in individuals coming from a country with high prevalence of specific mutations (e.g. Lithuania for PROP1 gene) or in cases with a specific hormonal, neuroradiological or extra-pituitary phenotype. By contrast, the genetic screening of pituitary transcription factors should not be part of routine work-up for Western-European sporadic patients with MPHD, as also suggested by other authors [15–17].

## Conclusions

1) In only few patients with congenital MPHD it is possible to detect a causative gene mutation; 2) therefore, it is fundamental to define some criteria for selecting the patients who should undergo genetic analyses; 3) such inclusion criteria should be based on the overall evaluation of hormonal clinical and neuroradiological phenotype; 4) it is crucial to consider whether the cases are sporadic or familial, since the probability of finding a causative gene mutation is distinctly higher in familial cases; 5) for PROP1 gene it is also important to consider the geographical origin of the patients, because this mutation is much more frequent in some ethnic groups.

## Abbreviations

APA: Anterior pituitary agenesis
GHD: Growth hormone deficiency
MPHD: Multiple pituitary hormone deficiency
MRI: Magnetic resonance imaging

## References

[1] Murray PG, Dattani MT, Clayton PE (2016). Controversies in the diagnosis and management of growth hormone deficiency in childhood and adolescence. Arch Dis Child.

[2] Giordano M (2016). Genetic causes of isolated and combined pituitary hormone deficiency. Best Pract Res Clin Endocrinol Metab.

[3] De Rienzo F, Mellone S, Bellone S, Babu D, Fusco I, Prodam F (2015). Frequency of genetic defects in combined pituitary hormone deficiency: a systematic review and analysis of a multicenter Italian cohort. Clin Endocrinol.

[4] Scommegna S, Galeazzi D, Picone S, Farinelli E, Agostino R, Bozzao A (2004). Neonatal identification of pituitary aplasia: a life-saving diagnosis. Review of five cases. Horm Res.

[5] Arrigo T, Wasniewska M, De Luca F, Valenzise M, Lombardo F, Vivenza D (2006). Congenital adenohypophysis aplasia: clinical features and analysis of the transcriptional factors for embryonic pituitary development. J Endocrinol Investig.

[6] Navardauskaite R, Dusatkova P, Obermannova B, Pfaeffle RW, Blum WF, Adukauskiene D (2014). High prevalence of PROP1 defects in Lithuania: phenotypic findings in an ethnically homogenous cohort of patients with multiple pituitary hormone deficiency. J Clin Endocrinol Metab.

[7] Fofanova OV, Takamura N, Kinoshita E, Yoshimoto M, Tsuji Y, Peterkova VA (1998). Rarity of PIT1 involvement in children from Russia with combined pituitary hormone deficiency. Am J Med Genet.

[8] Halász Z, Toke J, Patócs A, Bertalan R, Tömböl Z, Sallai A (2006). High prevalence of PROP1 gene mutations in Hungarian patients with childhood-onset combined anterior pituitary hormone deficiency. Endocrine.

[9] Lemos MC, Gomes L, Bastos M, Leite V, Limbert E, Carvalho D (2006). PROP1 gene analysis in Portuguese patients with combined pituitary hormone deficiency. Clin Endocrinol.

[10] Lebl J, Vosáhlo J, Pfaeffle RW, Stobbe H, Cerná J, Novotná D (2005). Auxological and endocrine phenotype in a population-based cohort of patients with PROP1 gene defects. Eur J Endocrinol.

[11] Vieira TC, Boldarine VT, Abucham J (2007). Molecular analysis of PROP1, PIT1, HESX1, LHX3, and LHX4 shows high frequency of PROP1 mutations in patients with familial forms of combined pituitary hormone deficiency. Arq Bras Endocrinol Metabol.

[12] Turton JP, Mehta A, Raza J, Woods KS, Tiulpakov A, Cassar J (2005). Mutations within the transcription factor PROP1 are rare in a cohort of patients with sporadic combined pituitary hormone deficiency (CPHD). Clin Endocrinol.

[13] Riepe FG, Partsch CJ, Blankenstein O, Mönig H, Pfäffle RW, Sippell WG (2001). Longitudinal imaging reveals pituitary enlargement preceding hypoplasia in two brothers with combined pituitary hormone deficiency attributable to PROP1 mutation. J Clin Endocrinol Metab.

[14] Tkacenko N, Lasiene D, Jakstiene S, Basevicius A, Verkauskiene R (2009). Evaluation of pituitary imaging in patients with prop-1 gene mutation. Medicina (Kaunas).

[15] Choi JH, Jung CW, Kang E, Kim YM, Heo SH, Lee BH (2017). Rare frequency of mutations in pituitary transcription factor genes in combined pituitary hormone or isolated growth hormone deficiencies in Korea. Yonsei Med J.

[16] Fang Q, George AS, Brinkmeier ML, Mortensen AH, Gergics P, Cheung LY (2016). Genetics of combined pituitary hormone deficiency: roadmap into the genome era. Endocr Rev.

[17] Elizabeth M, Hokken-Koelega ACS, Schuilwerve J, Peeters RP, Visser TJ, de Graaff LCG (2018). Genetic screening of regulatory regions of pituitary transcription factors in patients with idiopathic pituitary hormone deficiencies. Pituitary.

